# Strong yet incomplete reproductive isolation in *Vermivora* is not contradicted by other lines of evidence: A reply to Toews et al.

**DOI:** 10.1002/ece3.7763

**Published:** 2021-06-17

**Authors:** Cody K. Porter, John L. Confer, Kyle R. Aldinger, Ronald A. Canterbury, Jeffrey L. Larkin, Darin J. McNeil

**Affiliations:** ^1^ Wildlife Biology Program Lees‐McRae College Banner Elk NC USA; ^2^ Biology Department Ithaca College Ithaca NY USA; ^3^ West Virginia Cooperative Fish and Wildlife Research Unit Division of Forestry and Natural Resources West Virginia University Morgantown WV USA; ^4^ Department of Biological Sciences University of Cincinnati Cincinnati OH USA; ^5^ Department of Biology Indiana University of Pennsylvania Indiana PA USA; ^6^ Department of Entomology The Pennsylvania State University University Park PA USA

## Abstract

Toews et al. assert that strong reproductive isolation in *Vermivora* is inconsistent with other lines of evidence. Here, we discuss how strong yet incomplete reproductive isolation is consistent with other results from this system.

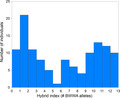

## CONFLICT OF INTEREST

The authors have no conflicts of interest to declare.

## AUTHOR CONTRIBUTION

**Cody Keith Porter:** Conceptualization (lead); Visualization (supporting); Writing‐original draft (lead); Writing‐review & editing (lead). **John L Confer:** Conceptualization (supporting); Writing‐original draft (supporting); Writing‐review & editing (supporting). **Kyle R. Aldinger:** Conceptualization (equal); Writing‐original draft (supporting); Writing‐review & editing (equal). **Ronald Canterbury:** Writing‐review & editing (supporting). **Jeffrey L Larkin:** Conceptualization (equal); Writing‐original draft (supporting); Writing‐review & editing (equal). **Darin J McNeil:** Conceptualization (equal); Visualization (lead); Writing‐original draft (supporting); Writing‐review & editing (equal).

“*It is no exaggeration to say that if no instances of uncompleted speciation were discovered [*. *. .] we would have to conclude either that evolution did not occur or that the formation of new species is instantaneous. What is a difficulty to the cataloguing systematist is a blessing to the evolutionist*.”

‐Dobzhansky T (1958) Species after Darwin. In: A Century of Darwin (ed. Barnett SA). Heinemann, London.

## 1 DISCUSSION

### 1.1 Plumage cannot be used to estimate hybrid ancestry in Vermivora

Toews et al. (2021) raise three main objections to our study wherein we document behavioral isolation and sexual selection against hybrid males between two Vermivora warblers (Confer, Porter, et al., 2020). Here, we respond to each of these critiques. While we agree with some aspects of Toews et al. (2021), we also contend that they misrepresent our manuscript, omit or misrepresent key pieces of the Vermivora and broader evolutionary literature, and offer a naïve conservation outlook on one of North America’s most imperiled songbirds. Nonetheless, we suggest that some of these disagreements are fruitful, as they highlight broader conceptual and methodological issues in the study of speciation. These issues are especially complex when studying the critical early stages of divergence, as recognized by Dobzhansky over 60 years ago.

The first issue Toews et al. (2021) raise is that we allegedly described primary hybridization as the mating of “genetically pure” *V*. *chrysoptera* and *V*. *cyanoptera*. Toews et al. assert that this is problematic because their recent work indicated that plumage phenotype does not predict hybrid ancestry in *Vermivora* (Baiz et al., 2020; Toews et al., 2016). However, this substantially misrepresents our manuscript. For the purposes of our study, we actually “…equate primary hybridization to the formation of a social pair between *phenotypes* of Golden‐winged and Blue‐winged warblers.” (Confer, Porter, et al., 2020, pg. 4). Our focus was, thus, on the frequency with which individuals with alternative plumage *phenotypes* form pairs, a point we emphasize many times throughout our manuscript. The two references to “genetic purity” in our manuscript both described the model of plumage inheritance in *Vermivora* envisioned by Parkes (1951), which we clearly described as being “insufficient” (Confer, Porter, et al., 2020, pg. 3) to explain the complexity of plumage inheritance in this system.

In essence, our study could be viewed as testing whether the six major genomic differences between *V*. *chrysoptera* and *V*. *cyanoptera* (which largely correspond to plumage differences; Toews et al., 2016) promote reproductive isolation. To this end, we focused on behavioral isolation and sexual selection against hybrids because past work indicates that plumage divergence in *Vermivora* underlies these reproductive isolating barriers (e.g., Leichty & Grier, 2006). We intentionally restricted the focus of our manuscript as such because we recognize that links between reproductive isolation and broader genomic patterns of divergence are not straightforward; especially in the early stages of speciation with gene flow, when reproductive isolation is incomplete, all of which characterizes *Vermivora* (Confer, Porter, et al., 2020; Toews et al., 2016).

For example, while the reproductive isolating barriers we documented may explain divergence in a handful of plumage‐associated loci (plumage variation is strongly correlated with an additive genotypic metric based on these loci; Toews et al., 2016), it appears there has been no “coupling” of reproductive isolation's effects to other, neutral genomic regions (Nosil et al., 2021). Theory indicates that, unless coupling occurs, we may expect to see extensive genomic homogeneity between diverging lineages, *even if reproductive isolating barriers eliminate all but a small amount of gene flow* (Barton, 2020; Flaxman et al., 2014; Nosil et al., 2021). This may be especially true when the trait(s) underlying reproductive isolation are oligogenic (Butlin & Stankowski, 2020) and strong selection drives divergence (Winker, 2021) which appears to be the case in *Vermivora*. Thus, the homogeneity of *V*. *chrysoptera* and *V*. *cyanoptera* genomes and the difficulty of assessing hybrid ancestry in this system is not evidence against the strength of the reproductive isolating barriers we documented. Instead, the genomic data may simply indicate that reproductive isolating barriers associated with a small number of loci have not “scaled up” to cause broader, neutral genomic differentiation between these lineages (Butlin & Stankowski, 2020). We stress that such patterns are widely expected early in speciation (Stankowski & Ravinet, 2021), when neutral processes driving genome‐wide divergence lag behind the strong diversifying effects of selection at a subset of loci (Winker, 2021).

### 1.2 Extra‐pair paternity is very high in Vermivora, likely facilitating hybridization

The second issue Toews et al. (2021) raise is that we do not accurately depict the potential impact that extra‐pair paternity may have on estimates of behavioral isolation. We partly agree with this criticism, namely, the point by Toews et al. (2021) that extra‐pair copulations (EPCs) need not be disassortative to bias our estimates of behavioral isolation as we originally stated. As Toews et al. (2021) correctly point out, even EPCs that are random with respect to phenotype *could,* under some circumstances, lead to us overestimating the strength of behavioral isolation.

However, the available evidence does not suggest that EPCs are likely to have biased our estimates of behavioral isolation. In the most comprehensive evaluation of extra‐pair paternity in *Vermivora* to date, Vallender et al. (2007) documented 53 cases of extra‐pair paternity where the phenotypes of the female, social male, and extra‐pair male could be determined (there were 148 cases of within‐pair copulations that could be confidently determined). In only three cases (1.5% overall) did a female's extra‐pair mate deviate from her social mate's phenotype in a way that increased disassortative mating (two cases of a hybrid female mating with a Golden‐winged Warbler and one case of a Golden‐winged Warbler female mating with a hybrid. Importantly, there are features of the population in Vallender et al. (2007) which make it likely that the potential for EPCs to bias estimates of reproductive isolation are at or near an upper bound. First, this population predominately consisted of *V*. *chrysoptera* and hybrids (Vallender et al., 2007); *V*. *chrysoptera* is especially likely to mate with hybrids, possibly because of their relatively high plumage similarity (Confer, Porter, et al., 2020). Second, the percentage of individuals with different phenotypes at this location was heavily skewed (*chrysoptera*: 74%, hybrids: 24%, *cyanoptera*: 2%). Large asymmetries in relative abundance such as this have long been known to facilitate heterotypic mating (“Hubbs principle”; Hubbs, 1955). The predominance of “assortative EPCs” even under these conditions suggests that *Vermivora* may be like other systems where assortative extra‐pair mating has been documented (e.g., Turbek et al., 2021).

Furthermore, Vallender et al. (2007) found that 72% of EPCs involved the male spatially closest to the female's breeding territory. Given the well‐documented differences in nesting habitat between sympatric *V*. *chrysoptera* and *V*. *cyanoptera* at multiple spatial scales (Confer & Knapp, 1981, Frech & Confer, 1987, Confer et al., 2003, Confer et al., 2010, Patton et al., 2010, Crawford et al., 2016, Wood et al., 2016), most EPCs are likely to involve males and females of the same phenotype, even if EPCs are random with respect to plumage phenotype. Thus, differences in nesting habitat may act as an automatic magic trait (Servedio et al., 2011) in *Vermivora*, which will promote divergence even if the scope for gene flow is large (Kopp et al., 2018). While we agree with Toews et al. (2021) that more work is needed on extra‐pair paternity and its consequences for reproductive isolation in *Vermivora*, the available evidence does not suggest that EPCs are “likely” to have biased our estimates of reproductive isolation.

### 1.3 The findings of strong total reproductive isolation are not compatible with previous work

Toews et al. (2021) contend that our findings (high behavioral isolation and moderate sexual selection against hybrid males) are inconsistent with long‐term data on *Vermivora* hybrid zone dynamics. Toews et al. (2021) emphasized an 8‐year study by Bennett et al.  (2017) that showed significant temporal change in the percentage of different *Vermivora* phenotypes within a population as being incompatible with strong reproductive isolation. Indeed, Toews et al. (2021) state that Bennett et al. (2017) documented “…consistent directional changes…” in the percentage of *V*. *cyanoptera*, *V*. *chrysoptera*, and, most importantly, hybrid phenotypes during their study. Toews et al. (2021) conclude that “These dynamics are not consistent with a scenario of high reproductive isolation between these forms…”.

**FIGURE 1:**
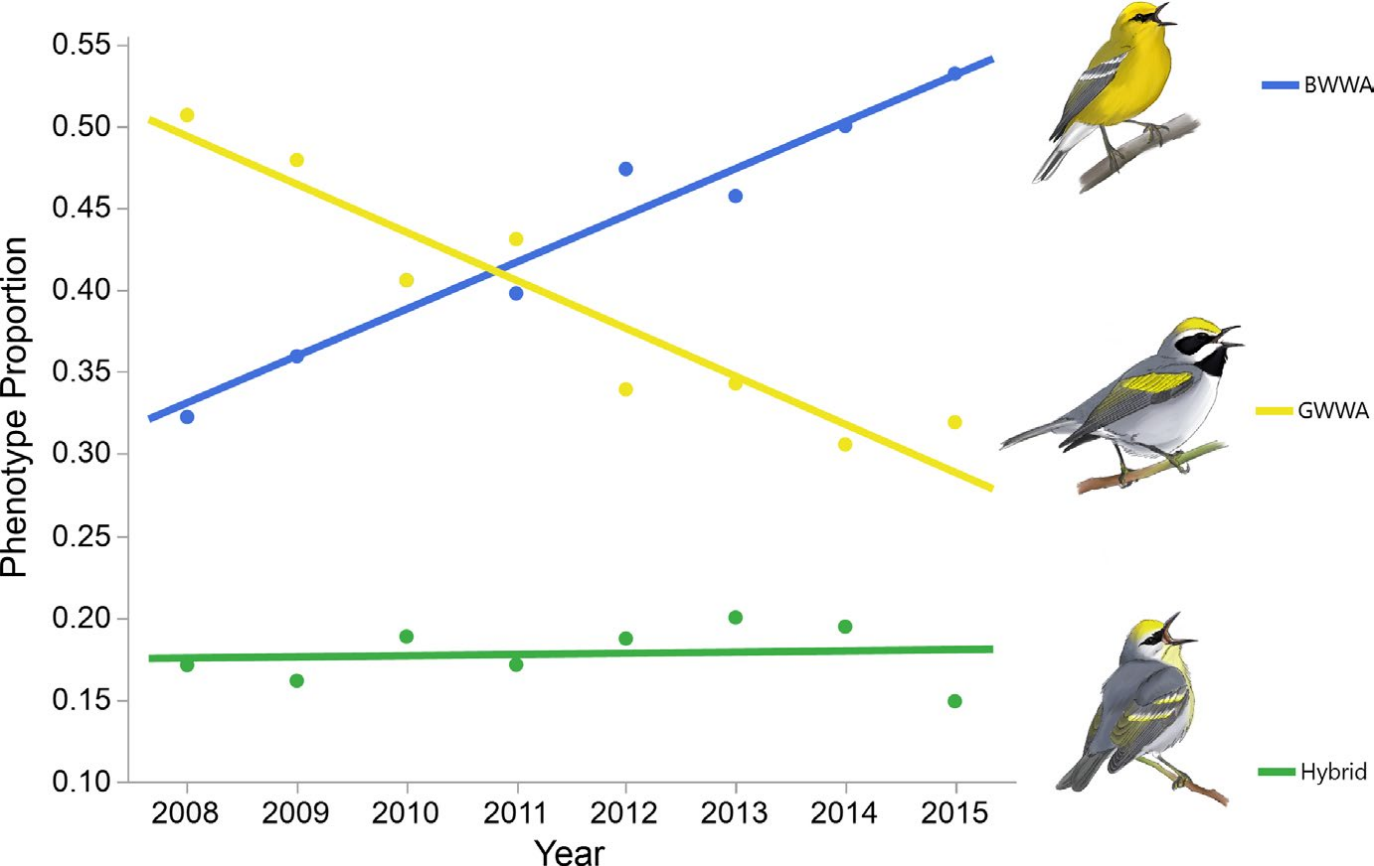
Over a seven‐year period at a site in northern New York, Bennett et al. (2017) found that the proportion of Blue‐winged Warbler phenotypes significantly increased, Golden‐winged Warbler phenotypes significantly decreased, and hybrid phenotypes remained constant. Figure reproduced from data in Bennett et al. (2017) with the permission of R. Bennett. Warbler illustrations by D. J. McNeil

The actual data in Bennett et al. (2017) paint a different picture. While Bennett et al. (2017) did find a significant increase in the percentage of *V*. *cyanoptera* phenotypes over time (*cyanoptera phenotypes* = −57.29 + 0.029*year, *p* < .001) and a significant decrease in *V*. *chrysoptera* phenotypes (*chrysoptera* *phenotypes* = 59.89 – 0.030*year, *p* = .0003), there was no directional change in the proportion of hybrid phenotypes (*hybrid phenotypes* = −2.612 + 0.001*year, *p* = .65). Indeed, there is no evidence of the “…intermediate stage involving substantial hybridization…” that Toews et al. (2021) refer to (Figure 1). We note that the pattern observed by Bennett et al. (2017) has been found in other areas of sympatry (e.g., Aldinger, 2018).

We argue that the data in Bennett et al. (2017) and elsewhere are more consistent with a scenario where one form (*V*. *chrysoptera*) is primarily declining due to factors other than an alleged lack of reproductive isolation. For example, *V*. *chrysoptera* must migrate substantially longer distances to the breeding grounds than *V*. *cyanoptera* (Bennett et al., 2017; their preferred explanation for the pattern of phenotypic change they document), experience a relatively higher incidence of building collisions during migration (Longcore et al., 2013; Loss et al., 2014), and have lower relative clutch sizes in areas of sympatry (Confer et al., 2003). By contrast, *V*. *cyanoptera* has a greater tolerance of habitat degradation (Crawford et al., 2016) and more generalized habitat use (Confer & Knapp, 1981) relative to *V*. *chrysoptera,* which use earlier stages of vegetative succession (Confer et al., 2020). Indeed, previous work has shown that vegetative succession over a similar time frame to that in Bennett et al. (2017) can lead to dramatic *V*. *chrysoptera* declines in areas without *V*. *cyanoptera* or hybrids (Martin et al., 2007; Otto & Roloff, 2012). Any of these factors combined with strong reproductive isolation better explains the data in Bennett et al. (2017) than the scenario of no reproductive isolation laid out by Toews et al. (2021), especially given the lack of change in the proportion of hybrid phenotypes over time.

Toews et al. (2021) also note that “…the vast majority of the reproductive isolation in Confer, Porter, et al. (2020) is attributed to pre‐mating isolation…”, which they argue is unlikely to maintain isolation in hybrid zones based on recent simulation studies (Irwin, 2020; Pulido‐Santacruz et al. 2018). In our manuscript, we emphasized that, although the numerical estimate of behavioral isolation (premating) is greater than postmating isolation (sexual selection against hybrid males), postmating isolation may be more effective at reducing gene flow, as suggested by Pulido‐Santacruz et al. (2018) and Irwin (2020). Indeed, Irwin (2020) demonstrated that even a 10% reduction in hybrid fitness alone is sufficient to maintain a narrow hybrid zone. Strong (10x) assortative mating combined with this relatively weak reduction in hybrid fitness maintains an even narrower zone (Irwin, 2020). On average, we found a 26% reduction in the pairing success of phenotypically hybrid male *Vermivora* relative to both parental forms and only 1.2% of birds with a “pure” phenotype paired with an individual of the alternative phenotype (i.e., strong plumage‐based assortative mating; Confer, Porter, et al., 2020). Thus, our data appear to fall well within the parameters for a stable hybrid zone according to Irwin’s (2020) simulations.

Finally, Toews et al. (2021) argue that strong behavioral isolation and sexual selection against hybrids are inconsistent with *Vermivora* genomic data which show evidence of historical introgression and homogenization of all but six loci, mostly associated with plumage divergence (Toews et al., 2016). However, if the behavioral isolation and sexual selection against hybrids we documented are associated with plumage divergence (as seems likely, based on experiments by Leichty and Grier, 2006), we might expect to see exactly the patterns found in *Vermivora* genomes, especially if divergence was relatively recent with ongoing gene flow throughout (as indicated by Toews et al., 2016). Under this genomic “islands of divergence” scenario, genomic regions associated with divergent selection and/or reproductive isolation (i.e., plumage differences) should be resistant to the homogenizing effects of gene flow and stand out in contrast to the rest of the genome (Nosil & Feder, 2012), as has been documented in other systems (e.g., Marques et al., 2016; Tavares et al., 2018). Indeed, *Vermivora* are just one of a growing number of systems where strong reproductive isolating barriers are present, yet genome‐wide divergence is weak (e.g., Karrenberg et al., 2019; Parchman et al., 2016, 2018; Sambatti et al., 2012; Turbek et al., 2021).

**FIGURE 2:**
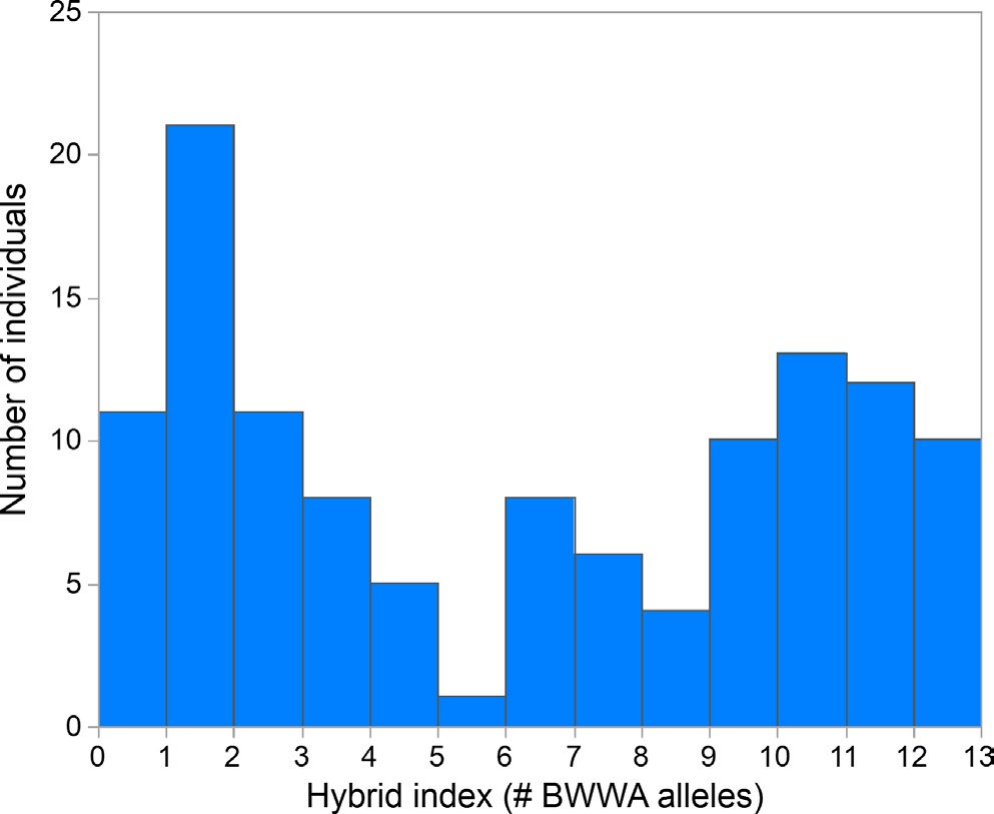
The distribution of hybrid index scores (number of *V*. *cyanoptera* alleles at the six most divergent loci; Toews et al., 2016) based on 120 *Vermivora* individuals sampled at a hybrid zone in northern New York in 2015. Bayesian normal mixture modeling found strongest support for a trimodal distribution of hybrid index scores and more support for a bimodal distribution than a unimodal distribution of hybrid index scores. These results hold if either of the two divergent regions on the Z chromosome are excluded from the analyses (thus accounting for potential linkage and non‐independence of these regions). This suggests there is strong yet incomplete reproductive isolation between *V*. *cyanoptera* and *V*. *chrysoptera* (Jiggins and Mallet, 2000, Stankowski & Ravinet, 2021). Importantly, the proportion of individuals with different phenotypes in extensive field surveys by Bennett et al. (2017) at this site in 2015 (cyanoptera: 0.53, chrysoptera: 0.32, hybrids: 0.15) is similar to the proportions in the genotypic samples of Toews et al. (2016) (cyanoptera: 0.47, chrysoptera: 0.37, hybrids: 0.16), suggesting no sampling bias that would increase deviation from a unimodal distribution of hybrid index scores (i.e., a scenario of no reproductive isolation). Finally, we note that *V*. *cyanoptera* and *V*. *chrysoptera* have been sympatric at this site since the late 1980s (Bennett et al., 2017), meaning there has been ample opportunity for gene flow to erode associations among these alleles

Again, we stress that these patterns are not unexpected and that measures of genome‐wide divergence are unlikely to correlate with levels of reproductive isolation in the early stages of speciation (Stankowski & Ravinet, 2021). A more appropriate approach for determining the extent of reproductive isolation from genetic data is to look at the distribution of the hybrid index score (Jiggins and Mallet, 2000) within a hybrid zone. The hybrid index score is estimated for each individual from multiple unlinked, highly divergent loci (Jiggins and Mallet, 2000). If reproductive isolation is weak, the association of alleles among regions will decay, producing a unimodal distribution of hybrid index scores (Jiggins and Mallet, 2000, Stankowski & Ravinet, 2021). At the other extreme, if reproductive isolation is complete, a bimodal distribution of hybrid index scores is expected (Jiggins and Mallet, 2000, Stankowski & Ravinet, 2021). Trimodal distributions of hybrid index scores are expected in scenarios of strong yet incomplete reproductive isolation (Jiggins and Mallet, 2000, Stankowski & Ravinet, 2021). Analysis of such genotypic data in Toews et al. (2016) from 120 individuals in the *Vermivora* hybrid zone studied by Bennett et al. (2017) does not support a scenario of no/minimal reproductive isolation (Figure 2): Bayesian normal mixture modeling (Brewer, 2003) finds strongest support for a trimodal distribution of hybrid index scores relative to a bimodal (ΔAICc = 13) or unimodal distribution (ΔAICc = 59). We note that genomic sampling of *Vermivora* in this location occurred in 2015 (Toews et al., 2016), after nearly three decades of sympatry and thus potential for hybridization between *V*. *chrysoptera* and *V*. *cyanoptera*. Thus, our finding of strong yet incomplete reproductive isolating barriers in *Vermivora* is consistent with the genomic data Toews et al. (2021) use to argue against our results.

A point of clarification on the strength of reproductive isolation may be helpful for resolving some of the disagreements raised in these manuscripts. The average strength of reproductive isolation from the combined action of behavioral isolation and sexual selection against hybrids we documented (0.96, on a scale of −1 to 1, where 1 is complete isolation and −1 is complete disassortative mating) may be considered strong in some contexts. Given that much of the *Vermivora* narrative suggests free interbreeding between *V*. *chrysoptera* and *V*. *cyanoptera* (i.e., reproductive isolation ≈ 0; Parkes, 1951; Rhymer & Simberloff, 1996; Tobias et al., 2010; Toews et al., 2018), our results show a strong deviation from this expectation. Nonetheless, reproductive isolation of 0.96 also means there is more than enough gene flow to homogenize neutral genomic regions or those unlinked to regions underlying reproductive isolation and/or divergent selection (Barton, 2020). Therefore, in terms of capacity to cause genome‐wide divergence between lineages, the reproductive isolation we documented may also be considered weak.

### 1.4 How should we study speciation?

“*…isolating barriers must be a central object of work on speciation. While one can infer the presence of isolating barriers from genetic or phenotypic data this gives no information about which barriers were the first to arise or what evolutionary forces created them*.*”*

‐Coyne, JA, and HA Orr (2004) Speciation. Sinauer, Sunderland, MA.

Identifying and quantifying the reproductive isolating barriers between lineages in the early stages of divergence offers unparalleled opportunities to explore the evolutionary forces driving speciation (Coyne & Orr, 2004; Nosil, 2012; Sobel & Streisfeld, 2015). Because genomic divergence, phenotypic divergence, and reproductive isolating barriers can accumulate after speciation is complete, inferences about the mechanisms contributing to speciation become extremely challenging at later evolutionary stages (Coyne & Orr, 2004) when clearer divisions between taxa are apparent. The body of literature on reproductive isolating barriers between nascent lineages is large and has produced many valuable insights into the speciation process, such as the role of adaptation in speciation (Nosil, 2012; Schemske, 2010; Sobel et al., 2010) and the relative importance of prezygotic and postzygotic barriers (Campillo et al., 2020; Coyne & Orr, 1989, 2004; Karrenberg et al., 2019; Lackey & Boughman, 2017; Lowry et al., 2008; Sobel et al., 2010; Sobel & Streisfeld, 2015). It is in this spirit that we sought to evaluate which reproductive isolating barriers may be acting in *Vermivora* (Confer, Porter, et al., 2020).

Although we disagree with many of the points made by Toews et al. (2021) as outlined above, we nonetheless acknowledge the difficulties of studying reproductive isolation during the early stages of divergence. In the genomics era, many researchers are grappling with the meaning of reproductive isolation. Some researchers appear to equate the evolution of reproductive isolation (driven primarily by selection; Schemske, 2010; Sobel et al., 2010) with the evolution of genome‐wide divergence (dominated by neutral or nearly neutral processes; Kimura, 1983; Ohta, 2002). Yet in systems with incomplete reproductive isolation, the level of reproductive isolation between groups may be uninformative for understanding the barrier to gene flow at any one locus (Butlin & Stankowski, 2020). In systems such as *Vermivora*, where most of the genome is homogeneous between two forms (Toews et al., 2016), the barrier to gene flow at most loci is small yet is large at a few major‐effect regions. Likewise, measuring the barrier to gene flow at a given locus using population genetic approaches does not provide an estimate of reproductive isolation (Butlin & Stankowski, 2020). Thus, we believe a balanced approach involving both empirical estimates of reproductive isolating barriers and genomic analyses is needed to resolve the complexities of the speciation process. We suggest that field estimates of reproductive isolating barriers such as ours are an essential component of future speciation research efforts.

### 1.5 Conserving species “of difficulty to the cataloguing systematist”

We agree with Toews et al. (2021) that conservation concerns should not drive the “production of research that contradicts the preponderance of available evidence.” As we have discussed here, we see no conflict between our findings and any previous research mentioned by Toews et al. (2021). Moreover, the science of conservation biology is underpinned by the conservation ethic (Sagoff, 2007; Sodhi & Ehrlich, 2010) and, therefore, cannot be blind to the real‐world implications of hasty decision‐making. There are few examples comparable to *Vermivora*, where one species is a candidate for protection under the Endangered Species Act yet its status as a species is also being reconsidered. Given this largely uncharted territory and inconsistent effects of taxonomic changes on conservation (Morison et al., 2009), it would be dubious to reclassify *Vermivora* without thorough examination of many lines of evidence (Stanton et al., 2019). Genomics provide one valuable perspective but should not be the sole basis for species delineation. This last point is especially salient in the genomics era, given that the biological species concept (upon which most ornithologists base species delineation decisions) allows for incomplete reproductive isolation and low levels of gene flow (Butlin & Stankowski, 2020; Coyne & Orr, 2004; Mayden, 1997) that can homogenize genomes (Barton, 2020).

Moreover, although Toews et al. purport that “…range‐wide endangerment status [of *V*. *chrysoptera*] is unlikely…”, virtually all evidence suggests otherwise. Populations are declining range‐wide (−2.57% change/year; Sauer et al., 2019), in the Appalachian Mountains (−7.82%), and in most of the Great Lakes region: Michigan (−5.41%) and Wisconsin (−2.57%). In fact, of the states and provinces for which the North American Breeding Bird Survey provides population trend estimates (*n* = 20), 12 (60%) show significant decline, seven (35%) trend on decline, one (5%; Manitoba) trends on growth, and none (0%) show significant growth (Sauer et al., 2019). Indeed, despite the notion presented by Toews et al. that Manitoba represents a stronghold for the species, *V*. *chrysoptera* populations remain so small in the province (Fink et al., 2020) that population trend estimates are unreliable (Sauer et al., 2019).

We acknowledge that Confer, Porter, et al. (2020) could have clarified the inconsistent and multifaceted conservation challenges (i.e., local, state, federal, nongovernment) associated with *Vermivora* taxonomic treatment (Morison et al., 2009). Toews et al. are correct that federal regulatory frameworks in the USA and Canada allow for listing of taxonomic divisions lower than species. We note, however, that *Vermivora* would not be classified as “evolutionarily significant units” (ESUs) as Toews et al. indicate, given that this designation was developed and has only been used for listing Pacific salmonids (61 FR 4,722, February 7, 1996). The correct listing mechanism in the USA if *Vermivora* were to be taxonomically lumped would be distinct population segments (DPS; 61 FR 4,722, February 7, 1996). Furthermore, both ESUs (Waples, 1995) and DPSs (61 FR 4,722, February 7, 1996) are evaluated based on differences from other populations of the same taxon. Toews et al. emphasize “low isolation and high gene flow” in *Vermivora*, which is seemingly at odds with their assertion that *V*. *chrysoptera* and *V*. *cyanoptera* qualify as ESUs.

## Data Availability

No data were generated in the production of this manuscript.
